# Complementarity as Relativity: Bohr’s Relativistic Analogy

**DOI:** 10.3390/e28101064

**Published:** 2026-09-27

**Authors:** Gregg Jaeger

**Affiliations:** Quantum Communication, Computing, and Measurement Laboratory, Department of Electrical and Computer Engineering and Division of Natural Science and Mathematics, Boston University, Boston, MA 02215, USA; jaeger@bu.edu

**Keywords:** complementarity, relativity, indeterminacy, quantum mechanics, Copenhagen interpretation

## Abstract

Niels Bohr asserted both that (i) the properties of quantum systems are specifiable, in general, only in relation to mutually exclusive experimental arrangements which may be freely chosen by experimenters, and that (ii) observed physical behavior is objective. In a move that reduces the apparent tension between those two assertions, Bohr put forward an analogy between quantum complementarity (in effect, the dependence of property values on measurement apparatus configuration in quantum mechanics) and the principle of relativity (in effect, the dependence of property values on the inertial reference frame in special relativity). Here, Bohr’s analogy is considered in a theoretical context. The elements of quantum mechanics and special relativity involved are set side by side, the analogous elements are identified with their roles in the analogy spelled out, and a number of dissimilarities that impact the force of the analogy are indicated—for example, quantum value determination, in general, requires an uncontrollable interaction during measurement, whereas non-quantum relativistic value determination does not. These dissimilarities point to weaknesses in the analogy, which he did not highly develop, and suggest that any stronger such analogy involving the two theories must take these differences more fully into account.

## 1. Introduction

Niels Bohr emphasized the essential and primary role of the apparatus in the measurement of properties appearing in quantum mechanical events, which he called phenomena. Phenomena, he says, occur in accordance with the notion of complementarity [1]. Bohr offered various analogies involving quantum theory in his writings. Among them is an analogy between complementarity in quantum mechanics (QM), which involves incompatible measuring apparatus arrangements, and the relativity postulate in special relativity (SR), which involves inertial reference frames moving at different speeds.

Notwithstanding all differences, a certain analogy between the postulate of relativity and the point of view of complementarity can be seen in this, that according to the former the laws which in consequence of the finite velocity of light appear in different forms depending on the choice of the frame of reference, are equivalent to one another, whereas, according to the latter the results obtained by different measuring arrangements apparently contradictory because of the finite size of the quantum of action, are logically compatible. ([2], p. 291).

Both theories specify the physical values of properties relative to different physical perspectives: in QM, the values of different quantities are, in general, specified in relation to measurements involving essentially different arrangements of apparatus; in SR, the values of quantities indicated by measuring instruments, such as rods and clocks, are specified in different inertial frames of reference. What Bohr holds to be common between them is the presence of consistency despite the appearance of values that can differ from perspective to perspective within each. These two modern theories provide consistent descriptions at least in part because of the presence of the two types of relationality brought in by the foundational notions of the theories, relativity and complementarity, which he argues are analogous.

Bohr also notes that (i) the physical laws of each theory involve a fundamental constant—in the case of SR, the speed of light *c*, and in the case of QM, the quantum of action *h*—that regulates the significance of the relational way in which they specify properties, and (ii) these constants of nature appear in the laws of each theory along with the imaginary unit $i=\sqrt{-1}$,

[T]he representation of the coördination of space and time in the theory of relativity by a four dimensional manifold, as also the connecting of kinematic and dynamic quantities in quantum mechanics by non-commutative algebra, rest essentially on the old mathematical artifice of the introduction of imaginary quantities; in fact the fundamental constants, the velocity of light and the quantum of action, are introduced into the formalism as factors of the $\sqrt{-1}$, the one in the definition of the fourth coordinate, the other in the commutation laws of canonically conjugate variables. ([2], p. 292),

with these shared features being related to the facts that (iii) in both cases their laws encompass behavior beyond ordinary experience where there is no apparent inconsistency and (iv) the values of these constants determine the circumstances under which the physical events they describe may exhibit extraordinary behavior in which their respectively relativities are manifest.

Bohr often discusses these two theories together in his writings and emphasizes that both describe physics objectively despite any suggestion to the contrary arising from the relational character of value specification in each. He attributes no causal influence, beyond their choice of the apparatus from which they gain knowledge, on values obtained to the subjects who observe them. Thus, the perspectives involved in these theories are fundamentally physical even when they are chosen.

Notwithstanding all differences in the typical situations to which the notions of relativity and complementarity apply, they present in epistemological respects far-reaching similarities…in neither case does the appropriate widening of our conceptual framework imply any appeal to the observing subject, which would hinder unambiguous communication of experience. ([3], pp. 6–7)

Bohr’s analogy can be viewed as supporting the objectivity of quantum physics to which he attributed great importance. This view of quantum physics was also supported by Vladimir Fock [4], who saw the analogy as support for the objectivity of quantum mechanics.

Bohr’s analogy was previously critiqued by Max Jammer [5] in connection with what he calls “Bohr’s relational conception of state,” which itself has very recently been cast as an example of soft perspectivism [6]. These previous treatments are discussed here in Section 3. Understanding Bohr’s analogy involves attending not only to the notion of complementarity and the principle of relativity but also to the laws and mathematical formalisms capturing the physical quantities to which they pertain. These latter are described and compared in the next section, and various weaknesses in Bohr’s analogy having their roots in the differences between them are pointed out in Section 4. Finally, in Section 5, it is shown that the limitations of Bohr’s analogy between QM and SR as presented are sufficiently significant for it to be assessed as weak.

## 2. Bohr’s Analogy and Theoretical Elements

For Bohr, quantum mechanics and special relativity both involve values of physical properties that must be specified relative to a physical situation that is classically describable, enabling them to be objectively communicated upon measurement. The relevant perspective in QM is that of the class of apparatus arrangement required for such measurement and, hence, for property value specification. Although the arrangements for the measurement of different sets of properties can be mutually incompatible, the measurement results are rendered theoretically consistent from the point of view of complementarity via the commutation laws of QM (e.g., Equation (1)) that connect observables. Bohr relates complementarity directly to the measurement of properties by measuring apparatus, which provide physical perspectives, as follows.

Far from restricting our efforts to put questions to nature in the form of experiments, the notion of complementarity simply characterizes the answers we can receive by such inquiry, whenever the interaction between the measuring instruments and the objects forms an integral part of the phenomena. ([7], p. 4)

The last phrase is crucial. (The various statements regarding complementarity in the writings of Bohr can be found discussed in, e.g., [8,9,10,11,12].). In SR, each physical perspective can be defined by a set of ideal objects, such as an ideal rod and an ideal clock that enable measurements, that moves at a constant velocity and so corresponds to an inertial frame of reference, but inertial frames of reference can also be considered in the abstract. In SR, objects are required to obey the principle of relativity, namely, the principle that the laws of physics that govern them are the same in all inertial frames of reference.

At the end of his reply to EPR [1], Bohr provides a prelude to his presentation of the analogy of QM with SR as it appears in [2].

[N]otwithstanding all characteristic differences, the situations we are concerned with in these generalizations of classical theory present striking analogies which have often been noted. Especially, the singular position of measuring instruments in the account of quantum phenomena, just discussed, appears closely analogous to the well-known necessity in relativity theory of upholding an ordinary description of all measuring processes, including a sharp distinction between space and time coordinates, although the very essence of this theory is the establishment of new physical laws, in the comprehension of which we must renounce the customary separation of space and time ideas.

Bohr notes here that SR involves a combination of interrelated space and time concepts, and suggests a similar relationship between property notions in QM that one finds expressed in the complementarity point of view. He categorizes QM and SR as generalizations of classical theory in which the relationships between such fundamental physical ideas is altered by their relationship to measurement perspectives. In his terminology these new theories are not “classical.” They are today considered the core of “modern physics.” (Note that I do not consider relativistic quantum field theory here because RQFT synthesizes rather than analogizes elements of the two.).

The quantum mechanical law Bohr refers to in [2] as the fundamental commutation law in the statement of his analogy,

(1) $$\left[X_{j},P_{k}\right]=i\hbar \delta_{jk}I\;$$

(j=x,y,z;k=x,y,z), governs the basic observables of position, $\overset{\to }{X}$, and momentum, $\overset{\to }{P}$, associated with motion. The law of Equation (1) contains both the imaginary unit and a fundamental constant of nature, *h*, as do other commutation relations between quantum observables, such as the components of spin associated with different directions that is discussed further below. The “finite size of the quantum of action” *h* guarantees the non-commutativity of the Hilbert-space operators for observables that corresponds to the physical incompatibility of the apparatus configurations required for their respective measurement in general practice. This is in accordance with complementarity because the measuring apparatus is thus an integral part of any uniquely quantum phenomenon. This law precludes, in general, the precise joint attribution of property values in QM. One could also mention, although Bohr does not do so explicitly, that *h* is the same in all measurement contexts.

In SR, the (non-quantum mechanical) law of motion

(2) $$\overset{\to }{F}=\frac{d}{dt}\overset{\to }{p}=\frac{d}{dt}\left[{\left(1+{\left(\frac{|\overset{\to }{v}|}{ic}\right)}^{2}\right)}^{-1/2}\left(m_{0}\overset{\to }{v}\right)\right]=\frac{d}{dt}\left(\gamma m_{0}\overset{\to }{v}\right)\;$$

(where $m_{0}$ is the mass of the object of interest when at rest in the given inertial reference frame considered) that governs an object moving with velocity $\overset{\to }{v}$ in the frame considered, can also be seen (as written here) to contain both the imaginary unit and a fundamental constant of nature, *c*, as Bohr also notes. The specific values of the standard relativistic quantities of motion, position $\overset{\to }{x}$ and momentum $\overset{\to }{p}$, of an object take different values depending on the inertial reference frame in which it is observed. These values are parameterized by the speed $|\overset{\to }{v}|$ via the factor $\gamma ={\left(1-|\overset{\to }{v}{|}^{2}/c^{2}\right)}^{-1/2}$ that indicates the significance of *c*, the speed of light in vacuum, which in SR is postulated to be constant in all inertial frames of reference. Property values are nonetheless consistently attributed in any given inertial frame of reference. This law of Equation (2) can also be written without the imaginary unit as

(3) $$\frac{d}{d\tau }p^{\mu }=F^{\mu }\;,$$

where $p^{\mu }=m_{0}u^{\mu }=\left(m_{0}c,\overset{\to }{p}\right)$, $u^{\mu }=\frac{d}{d\tau }x^{\mu }$, $d\tau =\frac{1}{\gamma }dt$, and $F^{\mu }$ is the Minkowski force (μ = 0, 1, 2, 3), which exhibits the equivalence across in inertial reference frames, rather than the form of Equation (2) which is inertial-frame dependent. (Most fundamentally, the length of the space-time interval τ given by the Minkowski distance metric takes the same value in every inertial reference frame). One may also note, although Bohr does not, that the generators $K_{i}$ of Lorentz boosts also obey non-trivial commutation relations, for example,

(4) $$\left[K_{i},K_{j}\right]=-\epsilon_{ijk}J_{k}\;,$$

i=x,y,z;j=x,y,z;k=x,y,z, where the $J_{k}$ are the generators of spatial rotations. (Note that the imaginary unit *i* does not appear on the right-hand side of this equation, although it does when the generators considered are those for quantum mechanical spaces and then their commutation relation is even more similar to Equation (1)).

The empirical significance in QM of the limit where action-scales are large relative to *h* (and quantum and non-quantum predictions are supposed to come into correspondence) is similar to that of the limit in SR where the speed of the object in motion, from the perspective of the inertial frame considered, is negligible relative to *c* (so that relativistic effects become negligible). These limits are those of most ordinary experience, lending consistency to the history of physics despite the novelties of these modern theories which, for Bohr, are “rational generalizations” of previous physical theory, that is, modern as opposed to classical theories.

Bohr notes that, under the principle of special relativity, the laws of motion, in general, “appear in different forms depending on the choice of the frame of reference,” as is most evident when written as in Equation (2) due to corresponding differences of the value of γ, but “are equivalent to one another” in that they are valid in all of physical frames of reference, as is most evident when written as in Equation (3). (The physical description of motion must be indicated in a specific inertial reference frame. Always focused on the necessity of logical consistency, Bohr notes elsewhere that the “singular role of the speed of light signals representing an upper limit for any consistent use of the physical concept of velocity” [13], p. 70.). Likewise, as just noted, under complementarity in QM, the differences in the specification of values of two such quantities (connected with the essential differences in the arrangements required to measure them and the uncontrollability, in general, of energy-momentum exchange during measurement) is expressed via the operator commutators of observables appearing in its laws (e.g., Equation (1)), which are always valid.

The quantum laws express, among other things, the fact that quantities corresponding to two non-commuting observables never share a full set of eigenvectors in common; cf., e.g., [14], which also provides a measure different from the Heisenberg–Robertson indeterminacy relations that better captures the incompatibility, in general, of precise measurements of quantities with non-commuting observables. As illustrations of the sort of mutually exclusive measurement apparatus arrangements required to specify different quantities precisely, note that a critical element of an apparatus may, for example, be fixed in place or moveable as needed so as to detect an incident particle’s position or momentum, respectively, or located in one position or another to detect beam direction or beam interference.

Bohr’s analogy thus indicates a similarity in the ways that complementarity and the relativity principle preclude physical inconsistencies among physical event descriptions of the different physical perspectives that are involved in each of the two theories. The laws of QM are the same for different, mutually exclusive measurement apparatus arrangements they regard, just as the laws of SR are the same for differently moving inertial reference frames, which can also be defined by appropriate apparatus, such as rods and clocks used for specifying spatial and temporal quantities, even if they are typically considered in the abstract.

## 3. Previous Discussion of the Analogy

Although there has been little previous discussion of Bohr’s QM-SR analogy, that of Jammer, who calls it “Bohr’s comparison” of quantum mechanics to special relativity, is substantial. He provides the following reconstruction, where the two theories are viewed as similarly relational.

The role of inertial frames of reference, always equipped with the identical inventory of measuring rods and clocks, relative to which the physical phenomena are observed, is taken over in quantum mechanics, according to Bohr’s conception, by different experimental setups varying in their inventory of measuring instruments. And just as the choice of a different frame of reference in relativity affects the result of a particular measurement, so also in quantum mechanics the choice of a different experimental setup has its effect on measurements, for it determines what is measurable. ([5], p. 201)

Jammer suggests that Bohr takes SR frames of reference to be *always equipped with rods and clocks that affect measurement results*, defining a role supposed to be taken over by the measuring instruments of QM. (However, the manner in which values can be said to be affected by the physical perspectives provided by measuring devices is rather different in the two theories: an uncontrollable interaction is required in general in QM for the measurement of quantum microphysical systems but is not required by the frame of reference as standardly understood in SR. This point is taken up here in the next section.).

Jammer frames the “basic problem in quantum mechanics” as that of determining the probability of obtaining a given result by measuring a physical quantity Q for a system by means of an experimental arrangement A, rather than the probability that the system itself simply possesses the Q-value obtained. “Since the ‘state’ of the system S is the sum total (‘catalogue’)” of all these probabilities, “the state of the system depends not solely on S…but also on A.” Quantum measurement results thus depend not only on the system but also on the apparatus in a way that it did not according to classical physics. His analysis of Bohr’s analogy is carried out in these terms, which he takes to define “Bohr’s relational conception of state” [5], Sect. 6.5. Jammer considers Bohr to understand the state itself in QM as relational in a sense similar to that of any physical system subject to SR. “One may also regard the connection between Bohr’s relational conception of state with Einstein’s theory of relativity from the general point of view that in the historical development of physics attributes were gradually replaced by relations.” For Jammer, this historical development had already begun with “the transition from Aristotelian quantitative physics to Newtonian quantitative physics” ([5], p. 201).

In a move that places Bohr’s analogy in such an overarching historical perspective, Jammer goes on to consider hypothetically the formulation of “a theory of ‘perspectives’” where the term perspective denotes “a coordinated collection of measuring instruments either in the sense of reference systems as applied in the theory of relativity or in the sense of experimental arrangements as conceived by Bohr.” (ibid.) Jammer then turns to the notions of physical perspective in SR and QM as instances of such a general theory and, differently from his reconstruction of Bohr’s analogy itself, considers the perspective of SR as kinematical *rather than* instrumental:

A “relativistic frame of reference” may be regarded as a geometrical or rather kinematical perspective; Bohr’s “experimental arrangement” is an instrumental perspective. And just as the former relativized lengths or time intervals and deprived them of the attribute of being ‘possessed’ by the object, so did the latter with regard to dynamical variables such as position or momentum. ([5], pp. 201–202)

Significantly, he then notes that there is a “profound” difference between the ontological status of physical objects in the two theories because “the pointlike events which [SR] discusses are thought of as being real in every sense of the word” (ibid.), whereas Bohr held that “all new experience makes its appearance within the frame of our customary points of view and *forms of perception*” [italics by Jammer] [5], p. 203, taking this to express a more Kantian perspective on the nature of properties. Nonetheless, in conclusion, Jammer argues that “this disparity…does not affect the similarity of both theories as to the relational character of the objects of discussion.” ([5], p. 207).

Very recently, Covoni et al. have taken a perspectivist view of QM: “the idea that physical properties in quantum mechanics are not fixed independently of measurement contexts or observational setups, but rather emerge from specific interactions—without this implying a collapse into epistemic subjectivism” [6]. This view recognizes the conditioning of the description of a quantum object on the sort of specific measuring apparatus involved, but these authors are more critical than Jammer of Bohr’s analogy.

[W]e cannot fully follow Bohr’s analogy between the relation character of quantum mechanics and that of special relativity. While it is true, for instance, that the time measured on system Albert by two other reference frames—let us call them Werner and Niels—moving at different velocities relation to Albert is different, the analogy breaks down upon closer examination.

They see the analogy as failing, in particular, due to the lack of an invariant quantity in QM analogous to the proper time τ of SR. They insist on a stronger analogy than Jammer, who requires only that physical states are relational in both.

However, Covoni et al. provide no proof that there is no such invariant, and one may note, for example, that there is at least one analogue of the Lorentz subgroup of spatial rotations in the measurement of a single spin-1/2 system (or the qubit) as indicated, for example, by Caslav Brukner and Anton Zeilinger [15]. Brukner and Zeilinger identify this invariant as that corresponding to “information invariance,” a total conservation of probability across possible measurements of this system, which had previously been considered in [16] that discusses in detail the transformations involved in mathematically surveying all quantum measurement apparatus arrangements for standard quantum observables having Hermitian operators. This indicates a path toward a strong analogy—although one still limited in scope—between QM and *one* form of relativity, namely, Galilean relativity with respect to spatial reference frame perspective. Moreover, the lack of such an invariant analogous to τ under more general forms of quantum measurement has, as just noted, not been demonstrated.

## 4. Weaknesses of Bohr’s Analogy

Having surveyed the several elements of QM and SR involved in Bohr’s analogy here in Section 2 (after having reviewed Bohr’s statements of it in his papers of 1935–1936 in Section 1) as well as previous critiques of it in Section 3, let us consider the strength of Bohr’s QM-SR analogy in greater detail. First, note that Bohr begins the introduction of this analogy by noting that it is offered “notwithstanding all differences” in the foundations of the theories because this indicates that he acknowledges some dissimilarities between in the elements of these two “rational generalizations” of previous physical theory to be navigated. The question at hand is the extent to which these might preclude a strong such analogy, beginning with Bohr’s analogy itself.

First note that there are dissimilarities in the general character of the two theories on the most general level. SR is a principle theory in Einstein’s sense that specifies property values directly as simple tensors, whereas QM is a framework theory that, in general, specifies property values indirectly via a combination of Hilbert space structures rather than directly, and the relation of states to events differs accordingly. However, if one considers complementarity as a relativity principle, it could be argued that it is also a principle theory (see [17] for a discussion of such categorization of theories) and that the high-level differences of theoretical nature between QM and SR in themselves are not of great relevance to the question of the strength of Bohr’s analogy itself. But on other levels the theories bear dissimilarities that render the analogy, as presented, a rather weak one.

Consider now these specific and interrelated aspects involved in the analogy: (i) the physical perspectives, (ii) the transformations between different perspectives, (iii) the quantities that are unchanged (invariants) under the change of perspective, and iv) the compatibility of different physical perspectives (in the sense that the descriptions of different perspectives can be combined without contradiction, that is, are logically compatible in Bohr’s sense without invoking counterfactual assumptions). Note that (iv) is enabled by (ii) and (iii) as they relate to (i), namely, perspective. A strong analogy of QM to SR would involve an isomorphism with respect to every one of these aspects of the two theories, but one finds sufficient dissimilarities in each of those aspects that preclude this. The most significant aspect that lies at the core of the analogy that Bohr presents is that of physical perspective, as it was considered to be in Jammer’s reconstruction. But, perspective involves not only the configured apparatus that Jammer considered to condition the quantum state, that is, render states relational but also the corresponding degree of specification of property values, that is, the precision with which they are specifiable via those states and whether values are determinate.

Let us consider each of these aspects in succession, beginning with that of the differences in the content of the two sorts of physical perspective themselves (i.e., aspect i). One finds significant dissimilarity between the sorts of perspective in the two theories both as presented by Bohr and as reconstructed by Jammer. The physical perspective in QM, which Bohr takes to be one regarding phenomena subject to complementarity, involves value-indeterminacy. But in SR the values of all properties in all perspectives can be always determinate in SR (e.g., in its usually non-quantum mechanical application)—what Jammer considers being “real in every sense of the word.” The laws of QM determine property values only statistically. “The very fact that repetition of the same experiment…in general yields different recordings pertaining to the object, immediately implies that a comprehensive account of experience in this field must be expressed by statistical laws.” [3], p. 4. Indeed, this was a point of contention between Einstein and Bohr, in relation to which the Einstein asked the well-known question “…*ob der liebe Gott würfelt*”—whether God plays dice. (Einstein used this now famous phrase in a letter to Max Born: “The theory produces a good deal but hardly brings us closer to the secret of the Old One. I am at all events convinced that He does not play dice.” [18]) Einstein’s concern regarded the application of quantum probability to individual systems. This dissimilarity in perspective thus concerns physical events themselves as described by the two theories. QM functions by supplying the probabilities of future measurement outcomes, whereas SR involves definite, non-statistical properties that can be precisely predicted.

The differences in content of the perspectives in QM and SR relate to the differences in the physical nature of the transformations involved (aspect ii) even if, in certain instances, such as the spin-1/2 system in QM, may involve transformations of quantum states of different quantities that involve some of the same *mathematical* group structures as in SR. These differences are significant and transcend the identity of mathematical groups involved. Although the theories both impose mathematical symmetry requirements connected with changes of physical perspective, the transformations realizing these symmetries differ in physical character: the transformations of values between inertial reference frames in SR are passive in the sense that, for example, rods and clocks appear rescaled in a way that the quantities they are used to measure are *determinately* value-interdependent in the change of perspective between differently moving inertial reference frames, whereas the relevant transformations of apparatus configuration between measurements of different physical properties in QM are always active in the sense that that, in general, they alter not only the (expectation) values of properties, but whether they will appear (definitely) at all, despite the fact that in QM one can also consider changes of Hilbert-space vector eigenbases in the absence of measurement when considering the likelihoods of values counterfactually, that is, *in the absence of* a choice of the actual apparatus configuration.

With regard to the question of an invariant quantity (aspect iii) analogous to τ raised by Covoni et al., only the total conservation of probability distributed among possible standard quantum measurements has been identified as such. However, this might be viewed as of limited physical significance because it can be understood to follow from the simple requirement on the statistics of quantum measurements that they be able to be interpreted in terms of probability and because it is of merely Euclidean form.

Finally, the compatibility of different physical perspectives in the sense that the descriptions of different perspectives can be combined without contradiction, that is, are logically compatible in Bohr’s sense without invoking counterfactual assumptions, aspect iv, depends on the extent to which aspects i-iii are similar in the two theories, as these similarities are needed for (iv). And in Bohr’s formulation of the QM-SR analogy, they are at best weakly similar.

These differences are weaknesses of Bohr’s analogy that are rooted most fundamentally in the differences in the descriptions of and the specifications of physical events by the two theories. Events in SR take place at spacetime locations where systems happen to coincide and can provide all property values determinately, whereas in QM a precise value for a measurable property is, in general, not definite and is found only when the requisite actively measured value for the corresponding observable is the last value to have been determined among those for all observables belonging to the sets of non-commuting observables to which it belongs. In measurements of the non-quantum systems (non-quantum) SR describes, any differences of outcome that are found upon repetition under identical conditions can be controlled and/or accounted for by traditional sources of measurement error. All property values in SR may be jointly specifiable in all possible perspectives (for example, the spatial extent of a single clock can be used as a length measure while the clock also indicates time), whereas the events of QM involve sets of properties that occur only probabilistically, in general, and in accordance with the Heisenberg–Robertson indeterminacy relations which indicate limits of precision with which values can be jointly specified, and property values appearing in events are determinate, in general, only if an appropriately configured active measurement apparatus is physically present. These differences present significant challenges to any analogy involving the two theories.

One might inquire into whether there is a way to avoid some of these analogical weaknesses that appear inherent in the standard quantum-physical descriptions of events when they are so compared to special-relativistic ones, for example, through more subtle uses of the QM formalism. Bohr’s analogy might be less weak than it appears if, say, the effects of the uncontrollable physical change of energy-momentum during periods of measurement could be circumvented, so that it would be of secondary significance for the determination of property values in quantum events much as it is for non-quantum special relativistic events. For example, it might be thought that all system property values in QM could be precisely jointly specified for any given time by inferring values of non-commuting observables at instants when *no* interaction takes place—the hypothetical events considered for such instants were called “interphenomena” by Hans Reichenbach [19]. (One might suppose, for example, that it is only when a measurement is taking place that quantum properties are indeterminate.). That is, one might attempt such inferences by making use of the results of measurements of non-commuting observables at times other than the given instant of interest in order to circumvent the difficulty posed by impossibility of the joint measurement of non-commuting observables at that time. Simple, passive transformations of quantum perspective, namely, mere mathematical changes of Hilbert-space basis analogous to those between inertial reference frames could then be considered to relate precise property values of non-commuting observables. In that case, by mitigating the apparent difficulties regarding perspective (aspect i), the analogy might remain strong in the face of the above challenges involving the differing natures of events and their measurement in the two theories.

In an attempt to specify precisely more (or all) values of quantum properties at any given time *t*, one could imagine inferring them from the results of measurements performed at other times, as follows. To specify the values of a pair of non-commuting observables at *t*, one might imagine using the quantum states of two different times: one, the state $|\psi_{0}\rangle$ found in an earlier preparatory measurement completed at time $t_{1}<t$, to infer the value of one property at *t*, and the other, the eigenstate $|\psi_{m}\rangle$ found in a later measurement completed at time $t_{2}>t$ to infer the value of the other property at *t*, avoiding simultaneous measurements. (This situation is considered here because it is already well known from textbook examples that the remaining alternatives, those involving successive previous or later measurements cannot be used for this purpose.). That is, one could imagine combining a prediction of value made using $|\psi_{0}\rangle$ and a retrodiction made using $|\psi_{m}\rangle$ (via the unitary Schrödinger state evolution, which is invertible) to find values of both at the different, single interaction-free instant *t* of time in between $t_{1}$ and $t_{2}$. This might be thought to allow the joint specification of the values of two incompatible observables for such a time *t* despite the fact that these properties are not simultaneously measurable. However, QM does not enable a valid precise retrodiction of the second-measured quantity sought in the above situation in addition to the prediction of the value of the first. At best, QM allows statistical inferences of imprecise joint values of non-commuting observables (as discussed in the next section).

Although one may speculate as to the values of interphenomena, property values are not generally precisely inferable retrodictively according to QM because each measurement performed after a previous measurement of an observable that does not commute with the first-measured observable changes the system’s property values in an indeterministic way before their registration. As a consequence, the value found in the measurement of an observable completed at the above time $t_{2}$ cannot be used to determine the value at any $t<t_{2}$ of any observable not commuting with that last measured, which in this case is that observable measured at $t_{1}<t$ (despite the fact that that such a value could have been precisely *predicted* for time *t* had a measurement of it *had been* completed at $t_{1}$ instead of that which was measured). Even though the evolution of the quantum state vector *between* measurement processes is time symmetric, that evolution does not govern the entire period between the two times $t_{1}$ and $t_{2}$ in this situation because of the subsequent, incompatible measurement interaction between $t_{1}$ and $t_{2}$ (namely, that begun before and completed by $t_{2}$), despite the fact that measurement does not itself take place an intermediate time. There can be no retrodiction of such a property value precisely even in this situation because the non-trivial measurement completed at $t_{2}$ is an irreversible process that, in general, involves an uncontrollable change of state.

A retrodiction of a quantum-mechanically measured property value of the above sort can be made based of a standard quantum measurement only if the state evolution between the times corresponding the completion of the two measurements is a free or controllable one. (The repetition of a precise projective measurement is of the latter kind.). As just noted, the second measurement above, being that of an observable not commuting with the one previously measured, does not in general involve such an evolution because in general it includes an uncontrollable interaction, namely, that which takes place before the completion of second measurement, that is, before $t_{2}$. Moreover, if an indeterministic, irreversible change has taken place by the time of completion of second measurement and is ignored in the inference, two different (quantum-complete) states for the same system for the unique time *t* would be calculated (that of the prediction and that of the retrodiction).

However, it is important to note that existence of a nonzero commutator is not tantamount to the inability of a given apparatus arrangement to find every possible pair of joint property values or uncontrollable disturbance. Rather, it captures the inability to measurement jointly observables for *all* such pairings—non-commuting observables can share *some* common eigenvectors. And the post-measurement state depends on all aspects of the process that accomplishes measurement, not only on those enabling measurement of the observable involved—that is, state preparation and measurement differ, in general. For example, a measurement may be destructive, in which case the system no longer exists as such after it is measured. One can also consider the behavior of a system and a measurement apparatus together with the interaction modeled unitarily, but one then runs into the quantum measurement problem (in which case there are, in general, no definite information-yielding measurement outcomes at all) and some addition mechanisms must be brought into play, which typically involve ad hoc elements related to the greater environment beyond the apparatus. There have been significant advances in quantum measurement theory relatively recently that might offer more subtle joint property specification, but these include non-standard, unsharp joint measurements (cf., e.g., [20,21] and references therein). The existence of such extensions of quantum measurement does not serve as reinforcement for Bohr’s QM-SR analogy, as presented, against the above weaknesses because they do not alter the inherent general lack of precise joint precise value specifiability in general in QM that underlies the dissimilarities of the two theories.

This leaves open the question of whether a related approach to a QM–SR analogy might be offered wherein there is greater similarity in the aspects (i)–(iv) identified above, say, by incorporating a different definition of perspective involving restricted or statistical sets of measurements that may avoid the difficulties faced by Bohr’s formulation.

## 5. Complementary in Interferometry

It was seen that, even for a time in which no measurement takes place, there are in general two different inferences needed to accomplish the desired perfectly precise joint valuation that require mutually exclusive sorts of data in order to be made. This cannot be circumvented. Consequently, the values for non-commuting observables of the system cannot, in general, both be specified at any single time even indirectly. Nonetheless, helpfully, later investigators, the first being William Wootters and Wojciech Zurek, have used standard QM methods to find best estimates of joint prior values of properties of non-commuting observables using a single measurement apparatus, providing insight into the limits of the joint specification of property values in QM [22]. This was carried out for quantum path determination and interference visibility in double-slit interferometry, and serves here as a more satisfying method for confronting the limitations of precision imposed by complementarity.

Although Bohr must have been aware that double-slit-type arrangements are capable of exhibiting different beam-states and interference visibility levels, it was only later, after his statement of the analogy, that this was shown explicitly. As the configuration of such an apparatus is altered, its measurements provide a full range of numerically complementary values of beam-path determination and interference visibility. These configurations, in effect, interpolate between those of the sort of two extreme apparatus of the kind that Bohr and Einstein had discussed—and had related to inferences as to particle position and momentum—prior to Bohr’s presentation of the analogy. The values of the measured properties preserve what Bohr called the logical compatibility of results but are, in general, jointly estimable with only *limited* precision.

Wootters and Zurek even derived a mathematical expression exhibiting the implication of measurement complementarity for these quantities in a double-slit apparatus. They did so by “assuming that the Heisenberg uncertainty principle holds,” and asking, “exactly to what extent the interference pattern is smeared out if we insist on determining the path of each photon with a given accuracy” in a double-slit arrangement [22], which allows for the partial determination of the path of the incoming beam while a less than full interference pattern appears on a final screen, and vice-versa, over a wide range of apparatus configurations. (Note that the Heisenberg uncertainty relations are derivable from the law of Equation (1).). They showed that two quantities, which correspond to conjugate Hilbert-space bases, namely, (1) retrodictive partial information about path and (2) interference pattern visibility are genuinely mathematically complementary across such intermediate configurations, demonstrating that less than full interference arises when beam direction is retrodictable with non-negligible precision. They argued that their results can be “stated in terms of an inequality, which sets the limit on the amount of retrievable information about the photons’ paths (photon-particle) for an assumed sharpness of the interference pattern (photon-wave),” namely, “(Information lost about the photons’ paths) ≥ (information H(S) lost in pure-state experiment giving the same interference pattern)” [22]. Here, H(S) is an entropic measure of information, where *S* is a measure of the sharpness of the interference pattern (ibid.)).

These results expose a significant weakness in Bohr’s analogy by exhibiting the limitations of the joint specification of system property values in QM by comparison to SR which has no such limitations, in particular, as regards the relation between property values which are precisely measurable only in alternative physical perspectives. But, they also indicate a similarity, albeit limited, between the QM and SR in that they consistently incorporate the different physical perspectives, providing objective descriptions of physical phenomena that lie outside of what has traditionally been considered ordinary human experience and can be understood to do so, at least in part, due to quantum complementarity.

## 6. Conclusions

Bohr argued that the properties of quantum systems are specifiable, in general, only relative to mutually exclusive experimental arrangements. He put forward an analogy between quantum complementarity—in effect, the dependence of property values on measurement apparatus configuration in quantum mechanics—and the principle of relativity—in effect, the dependence of property values on inertial reference frame in special relativity. Such relativity provides logical consistency among sets of corresponding physical values in both cases. However, close consideration of Bohr’s analogy shows that, as stated, it is rather weak due to a number of relevant dissimilarities and limitations of the available quantum state description by comparison with the non-quantum relativistic state description with regard to the specification of physical properties. This weakness may account for its not having been pursued further either by Bohr or others, such as Vladimir Fock [4], who also saw it as support for the view that quantum mechanics is an objective physical theory.

## Data Availability

The original contributions presented in this study are included in the article. Further inquiries can be directed to the corresponding author.
